# Sustainable Development Goals (SDGs), Public Health Expenditures, and Maternal and Child Mortality in Selected African Countries: Forecasting Modelling

**DOI:** 10.3390/ijerph22040482

**Published:** 2025-03-24

**Authors:** Yetunde Adegoke, Josue Mbonigaba, Gavin George

**Affiliations:** 1 School of Accounting, Economics and Finance, University of KwaZulu-Natal, West-Ville Campus Durban, Durban 4000, South Africa; mbonigaba@ukzn.ac.za; 2 Department of Economics, Federal University Oye-Ekiti, Oye-Ekiti 371104, Nigeria; 3 Health Economics and HIV and AIDS Research Division (HEARD), University of KwaZulu-Natal, Durban 4000, South Africa; georgeg@ukzn.ac.za

**Keywords:** SDGs target, maternal mortality, child mortality, PHEs, forecasting modelling, Africa, FQGLS

## Abstract

This study projects the performance of maternal and child mortalities in relation to the SDGs target (70 maternal deaths and 25 child deaths) by year 2030, based on three simulation scenarios of public health expenditures (PHEs). In essence, this study investigates the predictability of PHE in explaining maternal and child mortalities in a bid to confirm the possibility of meeting the SDGs target. The SSA is known to be facing critical health challenges; this study contributes to the problem underlying the health sector by forecasting PHEs in relation to goal 3 because the knowledge of correlation and threshold relationship between PHE and health outcomes, as seen in previous studies, may not be adequate to prepare the SSA countries towards achieving the SDGs target. This study uses Feasible Quasi-Generalised Least Squares as a baseline forecasting approach for 25 selected SSA countries. An increase in the PHE by 30 percent from the current level shows that only Botswana, Namibia, and South Africa will achieve the SDGs target of 70 maternal deaths, while Burundi, Cameroon, Central African Republic, Cote d’Ivoire, Eswatini, Lesotho, Mauritania, Niger, Nigeria, Tanzania, and Togo may have to bear more than 200 maternal deaths by 2030. In contrast, about 60 percent of the countries will achieve the SDGs target for child mortality. PHEs must meet the 30% increase forecasted for a reduction in mortality, being the benchmark that will enable the SSA region to achieve the SDGs target by year 2030.

## 1. Introduction

Sub-Saharan Africa (SSA) has not been on track to achieving the health-related Sustainable Development Goals (SDGs) [1]. Specifically, the region has been bedevilled with a high, chronic maternal mortality and child mortality, resulting in a low life expectancy at birth (LEB) of 62 years in 2020, which is below the world average of 73 years. However, low-income countries in South Asia, the Middle East, and North Africa, in the same period, recorded an LEB of 70, 64, and 74 years, respectively. Although the United Nations (UN) target for maternal mortality is 70 deaths per 100,000 births and 25 deaths per 1000 live births for child mortality by 2030 [2], countries in SSA have much higher rates because of limited public health expenditure (PHE) [3,4,5]. The economic profiles of these countries do not support achieving these targets because of the unfavourable macro-economic environment in the region. According to [1], improvement in health outcomes in SSA must be met with an enabling environment that is devoid of socio-economic disparities, political instability, and infrastructural deficits. The persistence of macro-economic problems will continually make the achievement of the SDGs target a mirage if not tackled with policy-driven recommendations.

The average PHE in SSA was 1.9% of Gross National Product (GNP) from 1995 to 2019, while in 2020, it was 2.02% GNP [6], which was insufficient, and more is required to achieve the SDG targets. Given the poor health outcomes caused by inadequate health funding and unfavourable macro-economic environments, this study saw the need to forecast whether the countries under review could achieve the SDG targets based on various PHE scenarios. Although several areas of health sector performance like factors that determine maternal and infant health outcomes [7], threshold effect of health expenditures on health outcomes [1,8], impact of health expenditures on economic growth [9], and productivity [10,11] have been researched, none has projected the required PHE levels that can position the SSA countries for the achievement of goal 3 of the SDGs. In addition, although several studies exist on PHE forecasting for developed countries and emerging economies [12,13] (specifically [14] forecast PHE for the Netherlands), none have focused on SSA. Thus, this study sought to contribute to the existing literature by forecasting maternal/child mortality based on specific levels of improvement in PHE to empower the region to achieve the SDGs targets of 70 maternal deaths per 100,000 women and 25 child deaths per 1000 live births by 2030. To the best of the researcher’s knowledge, this is the first study that has simultaneously predicted maternal and child mortality based on specific levels of improvement in PHE to achieve SDG targets 3.1 and 3.2

The results of this analysis might inform policymakers and stakeholders about the necessary resources and the required amount of PHE they need to commit to realising the SDGs targets. The specific aim of this study was to forecast whether the countries in SSA under study could achieve the maternal and child mortality SDG targets based on per capita PHE scenarios of 10%, 20%, and 30%. The estimation model was formulated based on the knowledge from Peacock and Wiseman [15], and it was applied to this study because increases in government spending were viewed as occurring in stages, which makes it reliable for generating our forecasts. The theory also applies to the analysis conducted in the study because increased government spending on health may be effective when gradual.

The out-of-sample result of the analysis reveals that a 10% increase in PHE will not bring any reduction in maternal mortality in any of the countries considered, but with the same 10% increase in PHE, there will be reduction in child mortality in South Africa and Senegal only; therefore, the countries are projected to achieve the SDG target of 25 child deaths per 1000 live births by 2030. Specifically, with the specified increase in PHE, child mortality is estimated to decrease to 21 in South Africa and 18 in Senegal by 2030.

Considering a 20% increase in PHE, amongst the countries analysed, only South Africa was projected to achieve the SDG target of 70 maternal deaths per 100,000 by the year 2030; specifically, the maternal mortality in South Africa will be around 62. Meanwhile, a 20% increase in PHE will make the following countries in SSA achieve the SDG target for child mortality: Burkina Faso, Burundi, Cameroon, Eswatini, Gambia, Namibia, Rwanda, and Tanzania.

Considering a 30% increase in PHE, only Botswana and Namibia would be able to achieve the SDG target of 70 maternal deaths per 100,000 by 2030. In addition, Burundi, Cameroon, Central African Republic, Côte d’ Ivoire, Eswatini, Lesotho, Mauritania, Niger, Nigeria, Tanzania, and Togo might experience more than 200 maternal deaths by 2030, and Nigeria would still have more than 500 maternal deaths. Botswana, Sudan, and Togo will achieve the SDGs child mortality target with a 30% increase in PHE.

Following the introduction of this study, Section 2 presents the literature review, while Section 3 discusses the materials and methods. Section 4 discusses the results in light of the research objectives. The study concludes with a summary in Section 5.

## 2. Literature Review

Ref. [16] predicted out-of-pocket (OOP) PHE in Rwanda using machine learning techniques to identify the best model for this type of health funding. In Rwanda, the OOP PHE was 24.46% of health spending in 2000, increasing to 26% in 2015. Moreover, an integrated living condition survey was conducted during the analysis with about 14,580 households in Rwanda. In Muremyi et al.’s study, TreeNet was found to be accurate in predicting the OOP PHE, which led to the recommendation that the government increase PHE because of the poor performance of OOP health spending. This recommendation supports the claim of inadequate PHE in SSA.

Ref. [17] study assessed Eastern and Southern African countries’ efforts to achieve the SDGs by capturing the impact of PHE on health outcomes in 18 of these countries with an emphasis on the role of governance. The study period spanned from 2001 to 2017, and Generalised Method of Moment (GMM) was the estimating technique adopted. The results revealed that PHE (total, public, and private) had a significant negative relationship with health outcomes (under-five and maternal mortality). In contrast, a positive relationship was documented between PHE and LEB. In addition, the study revealed that PHE had a higher impact on health outcomes than private health expenditure because of the largely poor population that depends solely on the government for healthcare in Africa. For instance, in South Africa, 82.6% of the population depends on PHE, making only 17 in 100 South Africans able to access private health insurance schemes [18]. Moreover, the WHO (2020) contends that OOP funding does not contribute to equitable progress in achieving universal health coverage. Ref. [19] also used OLS regression to assess PHE’s impact on under-five mortality in Cameroon. The results showed that PHE had a negative but insignificant effect on under-five mortality.

Ref. [20]’s research on PHE’s impact on LEB in Nigeria from 1979 to 2019 employed a dynamic ordinary least square (OLS) method. The results revealed an insignificant positive relationship between PHE and LEB. Ref. [21] investigated the impact of macro-economic factors on health for the South African economy proxied by mental health conditions, metabolic risk factors, and non-communicable conditions of the adolescent and young adult population. The study adopted a two-stage least-squared (2SLS) model and discovered that a declining economy reduces cases of communicable conditions, while it exacerbates mental health conditions, metabolic risk factors, and non-communicable conditions. Ref. [22] investigated the macro-economic determinants of health outcomes in EU member states by analysing the impact of health financing on economic, behavioural, and country-specific characteristics on health outcomes. The study adopted fixed- and random-effect models on data that spanned from 2000 to 2018. The impact of the identified macro-economic variables was stronger on the child mortality rate than the life expectancy at birth. Public health expenditures, numbers of physicians, consumption of fruit and vegetables, decentralisation of the health system, and fixed-country effects were the determinants incorporated in the study. The study concluded that an increase in PHE alone may not be sufficient but must be complemented with improved education, healthy lifestyle, and investment in human resources to improve health services.

## 3. Materials and Methods

In this study, the forecasting analysis was conducted based on the macro-projection model, whereby the investigation extends to aggregate PHE, especially when making short-term projections with unperturbed trends and without structural breaks [23].

### 3.1. Estimating Technique

Generalised least squares are usually used to estimate the unknown parameters in linear regression. However, in this study, following the work of Westerlund and Narayan [24] that proposed the use of an FQGLS estimator to account for persistence of the variables in a forecasting model, the Feasible Quasi-Generalised Least Squares technique (FQGLS) was used because it would be consistent, asymptotically normal, and robust to heteroskedasticity. It also permitted the taking care of salient time series features of the data, such as unit roots, which reveal a systematic, unpredictable pattern, persistence effects, and an endogeneity bias. The forecasting model is stated to capture the deficiencies in the Lewellen [25] estimator because it accounts for only persistence in a forecast model.

### 3.2. Model Specification

The respective models for the expected negative relationships between PHE per capita (${Health}_{t}$) and maternal mortality (${Maternal}_{t}$) and child mortality (${Child}_{t}$) are as follows:

(1) $${Maternal}_{t}=\alpha +\beta {Health}_{t-1}+\varepsilon_{t}$$

(2) $${Child}_{t}=\alpha +\beta {Health}_{t-1}+\varepsilon_{t}$$

Based on the work of Salisu and Omotor [26] and the preceding specification, the Feasible Quasi-Generalised Least Squares approach (FQGLS) was adopted to address salient time series features of the data, including the unit root problem, persistence effect, and possible endogeneity bias. The updated specifications with double-log forms are as follows:

(3) $$\log\left({Maternal}_{t}\right)=\alpha_{1}+\beta_{1}\log\left({Health}_{t-1}\right)+\theta_{1}∆\log\left({Health}_{t}\right)+\gamma_{1}\log\left({Maternal}_{t-1}\right)+\varepsilon_{1t}$$

(4) $$\log\left({Child}_{t}\right)=\alpha_{2}+\beta_{2}\log\left({Health}_{t-1}\right)+\theta_{2}∆\log\left({Health}_{t}\right)+\gamma_{2}\log\left({Child}_{t-1}\right)+\varepsilon_{2t}$$

where the alphas ($\alpha_{1}$ & $\alpha_{2}$) are the constant terms, and the betas ($\beta_{1}$ & $\beta_{2}$) are the coefficients of interest that show the impact of PHE per capita on maternal and child mortality, respectively. In addition, θ and γ are the coefficients of the unit root and persistence accounted for in the models. ${Health}_{t}$ is PHE per capita at the present period, and ${Health}_{t-1}$ is PHE per capita in the previous period. ${Child}_{t-1}$ is the child mortality in the previous period, and ${Maternal}_{t-1}$ is the maternal mortality in the previous period. The model is stated dynamically, thus providing an opportunity for the previous level of health outcomes to explain some variations in the present level of health outcomes.

In the estimation of the models for the years 1995–2020, the data were derived from the literature and sourced from [6] for countries in SSA. Maternal mortality referred to pregnancy-related deaths per 100,000 women during and six weeks after delivery. This variable is one of the critical indices that can be adapted to proxy health outcomes. In this study, it was considered important because of the high level of maternal mortality recorded in SSA. Child mortality referred to the number of deaths per 1000 live births of children under five. PHE per capita (HEXPC), referred to as PHE, is the capital outlay on health provided by a country’s government, including the NHIS. This study measured the PHE as a percentage of the GNP per person, which is the domestic government PHE. The variable adopted as the most important determinant of health and the impact of PHE on health outcomes is well-established in the literature (WHO [17]).

The expected negative sign indicated by the theory in the relationship between PHE per capita and maternal/child mortality was sought. The estimated results were then utilised as the basis for in-sample and out-of-sample forecasting, with an analysis of an optimistic scenario that examined the likely impact of consistent growth in PHE per capita by approximately 10%. Consequently, during the out-of-sample forecasting analysis period (2021–2030), reductions in mortality were observed across the countries in SSA.

### 3.3. Forecasting Evaluation Method

The forecasting covers both the in-sample and out-of-sample periods. In order to capture the robustness of the model, about 50 and 75 percent of the total data observations were employed in the analysis, respectively, for the in-sample and out-of-sample evaluation. In this study, we favoured a recursive window approach to forecasting over the fixed-parameter approach because of the time-varying aspect of the estimate, which is adequate to capture the complex^1^ nature of the macro-economic system.

Single and pairwise processes were adopted in this study; specifically, the Mean Square Error (MSE) was adopted for the single forecast evaluation, while a Campbell–Thompson Statistic was preferred for the pairwise process.

In mathematical terms, the single forecast process can be stated as follows:

$$(\mathrm{MSE})-1/N\sum_{t-1}^{T}M\hat{M_{t}}-MM_{t}{)}^{2}$$

where $\hat{MM}$ stands for forecasted or fitted maternal mortality, while MM is the actual maternal mortality. N stands for the number of predictions used in computing the mean, with M$\hat{M_{t}}$ and M$M_{t}$ in the case of maternal mortality and C$\hat{M_{t}}$ and C$M_{t}$ in the case of child mortality.

The pairwise forecast process, you will recall, measures the Campbell–Thompson (C-T) statistic test, which denotes the out-of-sample R-squared (OOS_$R^{2}$). Therefore, the OOS_$R^{2}=1-(M\hat{S}E_{2}/M\hat{S}E_{1})$, where $M\hat{S}E_{2}$ and $M\hat{S}E_{1}$ stand for the MSE of the out-of-sample forecast emanating from the unrestricted and restricted models, respectively ($M\hat{S}E_{2}$ and $M\hat{S}E_{1}$).

Note: A positive C-T test statistic suggests that the unrestricted model performs better than the restricted model and vice versa for a negative C-T statistic test. Recall that the predictive model in Equations (3) and (4) as expressed in the FQGLS model is the unrestricted model, while, conventionally, two predictive models, like the historical average (HA) and autoregressive integrated moving average (ARIMA) models, are the restricted models.

The recursive window method allows us to deal with unexpected changes or structural breaks in time-varying parameters, and the addition of new observations and re-estimation are possible.

## 4. Results

Table 1 below shows the analysis of the relationship between health outcomes (maternal and child mortality) and PHE for each country in SSA. The preliminary purpose of this analysis was to determine whether a negative relationship existed between PHE, as hypothesised a priori, suggesting that an increased PHE would lead to reductions in maternal and child mortality.

### 4.1. Regression Results

Amongst the countries in SSA included in this study, a negative relationship was observed between health outcomes and PHE, as indicated in Table 1 below. The analysis covered 24 out of the 25 countries, with Mauritius being excluded due to its consistently low levels of maternal and child mortality since 2016 [6].

In Mauritius, child mortality has remained below 25 since 1995, and maternal mortality has been consistently below 70. The non-negative relationship between PHE and mortality in Mauritius is highlighted in bold in the table. In Mauritius, this relationship suggests that the country’s health outcomes are already at a relatively favourable level, and further increases in the PHE may not yield significant additional improvements in health outcomes. In addition, other factors apart from PHE may be contributing to the current level of child mortality observed in the country.

The regression results in Table 1 below provide strong evidence supporting the hypothesis that higher levels of PHE per capita would lead to better health outcomes in the selected countries in SSA. To further investigate the validity of this hypothesis, the researcher performed forecasting procedures to determine whether the observed relationship between higher PHE per capita and better health outcomes was likely to hold in the future. This would provide valuable insights for policymakers and stakeholders, enabling them to make informed decisions regarding resource allocation and prioritise investment in public health infrastructure and services to improve maternal and child health. These forecasting results will be explained later in this section.

Table 1 below shows the regression results for the relationship between health outcomes and PHE per capita.

### 4.2. In-Sample and Out-of-Sample Result

This study attempts to forecast the behaviour of maternal and child mortality in SSA in contrast to existing studies on impact analysis, which capture the long-run. This present study seeks to know the level of PHE which could achieve the SDG target over a short term.

**Figure 1:**
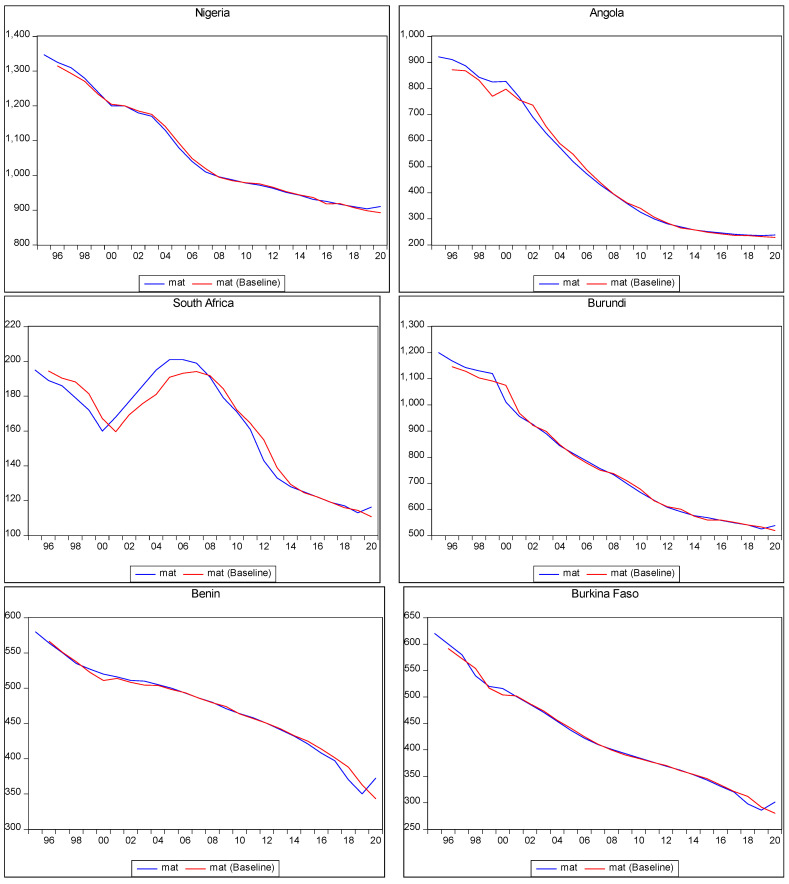

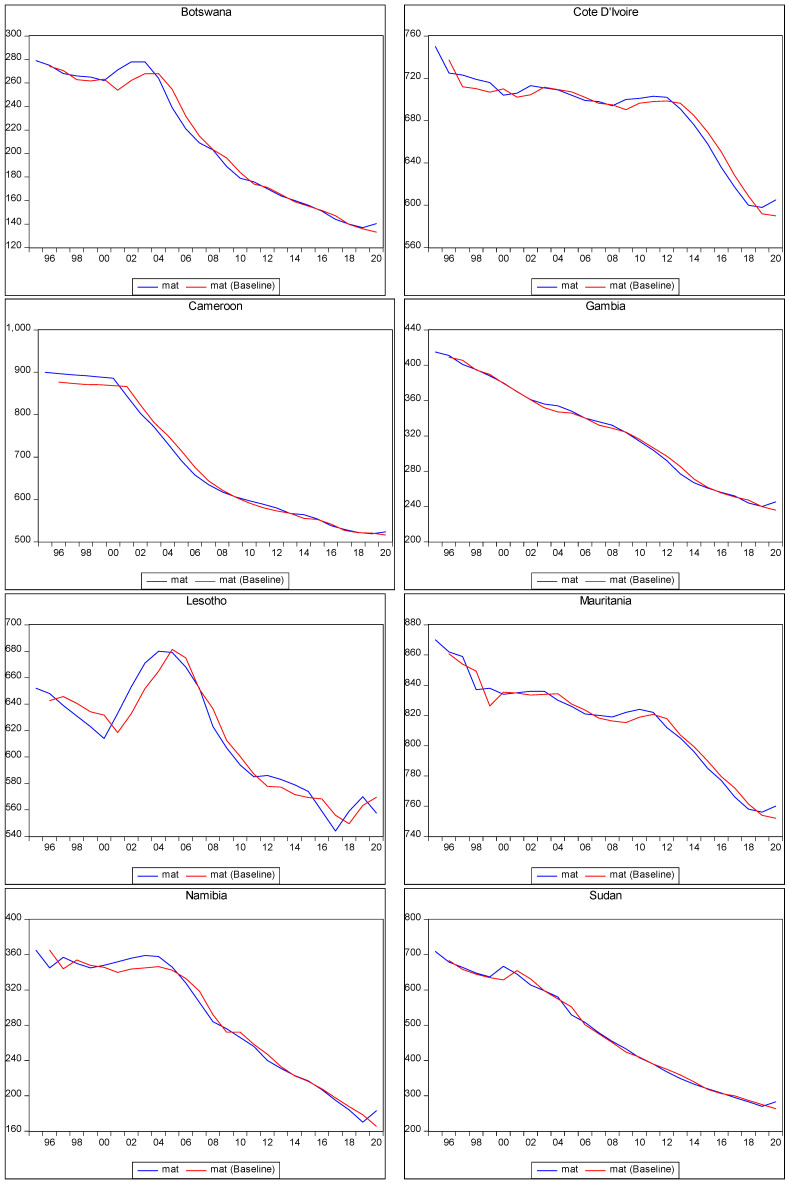

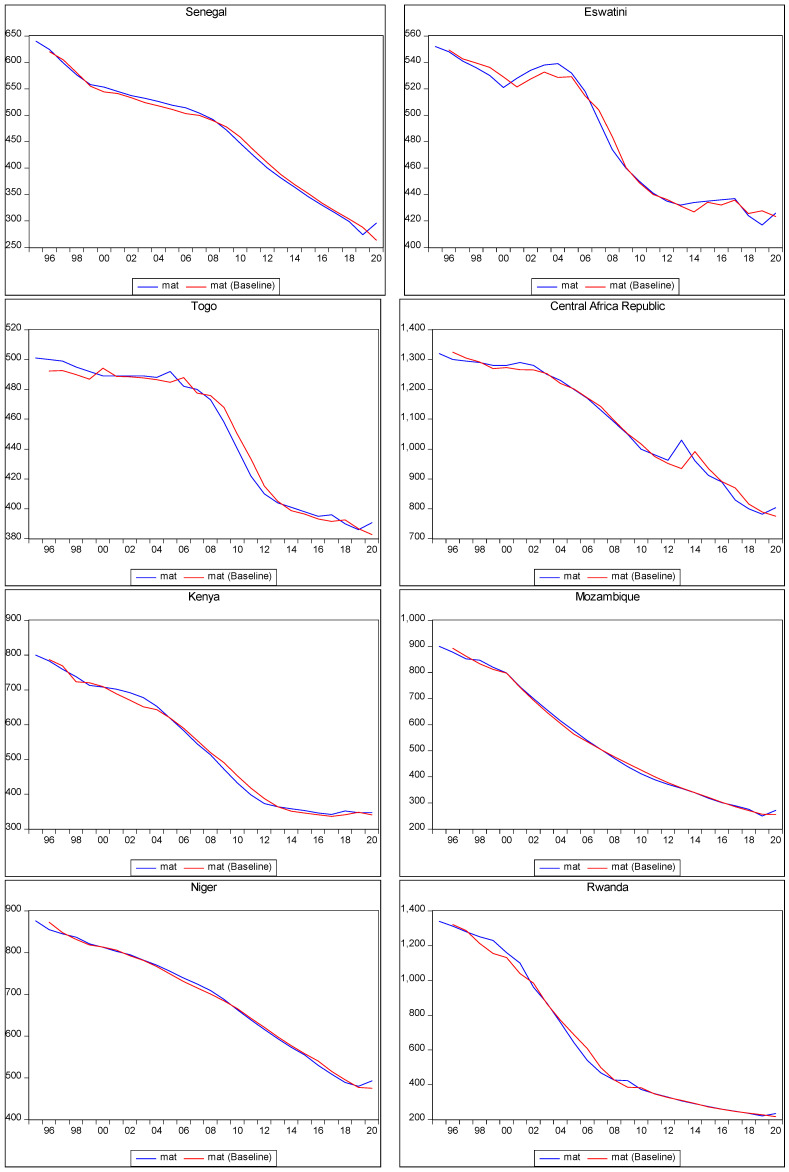

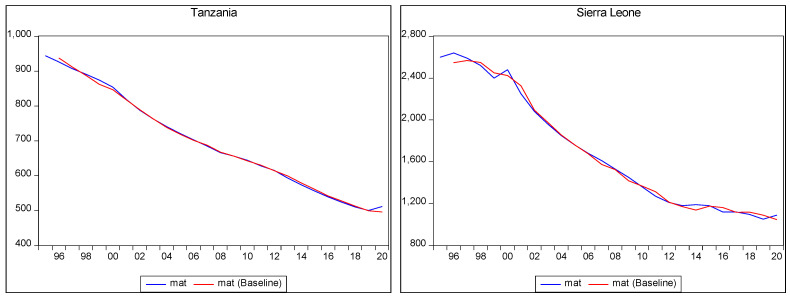
In-sample prediction of maternal mortality.

Figure 1 below reflects the in-sample forecast for maternal mortality. It demonstrates the movement path between the actual maternal mortality and the forecasted values within the given data period. The actual maternal mortality and the forecasted maternal mortality align closely, with minor variations. The x-axis represents years, while the y-axis represents maternal mortality per 100,000. In the figure, the red curve represents the observed or actual maternal mortality, while the blue curve represents the forecasted maternal mortality. The close alignment between these curves indicates that the model used for the in-sample forecast captures the historical patterns and variations in maternal mortality reasonably well.

The relationship depicted in Figure 1 below indicates a declining trend in maternal mortality over time amongst the countries in SSA. This suggests that maternal mortality rates decreased over the analysed period. The downward trend could suggest that efforts to address maternal mortality, including investments in PHE had some positive impact on reducing maternal mortality rates. However, despite the decreasing trend, maternal mortality remained a significant burden in these countries. This suggests that additional interventions and strategies would be necessary to further reduce maternal mortality rates and achieve desired SDG targets.

While Figure 1 below does not explicitly indicate the causal relationship between PHE and maternal mortality, it does suggest that investments in healthcare infrastructure and services, reflected in the declining trend of maternal mortality, have played a role in improving maternal health outcomes. It implies that PHE has likely contributed to the reduction in maternal mortality rates in the analysed countries in SSA, although it may not be the sole determinant.

The primary purpose of conducting in-sample forecasting is to assess the model’s ability to accurately predict outcomes based on the available data. In this case, the in-sample forecast provided insights into the model’s performance in estimating maternal mortality within the investigated period. By comparing actual and forecasted values, researchers can evaluate the model’s accuracy and reliability. This assessment helps build confidence in the model’s ability to make meaningful predictions and inform subsequent out-of-sample forecasting, which extends the predictions beyond the available data to estimate future outcomes.

Figure 1 below presents the in-sample forecast for maternal mortality in selected countries in SSA.

Figure 1 above presented in-sample forecasting of maternal mortality in various countries in SSA, as was explained above. However, the table below presents out-of-sample predictions of maternal mortality in a scenario of a 10% increase in the PHE of the countries in SSA.

Out-of-sample forecasting involves making predictions or projections for periods beyond the available data used to build the forecasting model. It aims to estimate future outcomes based on the relationships and patterns observed in historical data. In the context of maternal mortality forecasting, the out-of-sample forecast provided insights into the potential trends and outcomes for maternal mortality beyond the analysed data period. It allowed the researcher to explore the expected impacts of changes or interventions, such as a 10% increase in PHE, on maternal mortality rates. In this study, by utilising the forecasting model developed using in-sample data, the out-of-sample forecast estimated how maternal mortality may evolve in the future when certain conditions or factors change. In this case, the focus was on the effects of increased PHE on maternal mortality rates.

The out-of-sample forecast in Table 2 below displays the projected maternal mortality outcomes for 2021, 2025, and 2030, under the assumption of a 10% increase in PHE. The table indicates the potential trajectory of maternal mortality rates and offers insights into the progress countries may make towards achieving the SDG targets for maternal mortality.

The out-of-sample forecast demonstrates the potential outcomes for maternal mortality when PHE is increased by 10%. In cases where the relationship between PHE and mortality is non-negative, indicated as “Nil” in the table, the implication is that higher PHEs does not lead to a reduction in maternal mortality. Therefore, even with an increase in PHE, the expected impact on reducing maternal mortality is limited.

Considering the specific periods shown in the table, it becomes evident that none of the countries analysed were able to achieve the SDG target of 70 maternal deaths by 2030, despite the forecasted in-sample outcomes. This suggests that additional efforts beyond solely increasing PHE are required to address the complex factors influencing maternal mortality rates. Thus, the results underscore the need for comprehensive strategies and interventions beyond financial investment to combat maternal mortality and work towards achieving the SDG target.

Out-of-sample forecasts are, however, subject to inherent uncertainties and assumptions. The actual future outcomes may deviate from the projections due to various unforeseen factors and changes in the operating environment. Therefore, while out-of-sample forecasting provides valuable insights into potential future outcomes, it should be interpreted with caution, and policymakers should consider multiple factors and strategies beyond a single variable, such as PHE, to address maternal mortality and work towards achieving the SDG targets. It underscores the need for comprehensive strategies and interventions beyond financial investment to combat maternal mortality and work towards achieving the SDG targets.

Table 2 below presents the out-of-sample maternal mortality forecasting, which assumes a yearly 10% increase in current PHE per capita (real terms).

**Table 2:** Out-of-sample maternal mortality forecast with a 10% yearly increase in PHE. (Based on 75 percent observation).

Country	2021 Numbers Per 100,000	2025 Numbers Per 100,000	2030 Numbers Per 100,000
Angola	229	193	145
Burkina Faso	293	263	225
Benin	364	332	289
Botswana	136	120	103
Burundi	528	478	397
Cameroon	517	485	431
CAR	792	748	694
Côte d’Ivoire	596	558	502
Eswatini	426	414	380
Gambia	240	222	199
Kenya	337	284	211
Lesotho	556	521	456
Mauritania	755	734	703
Mozambique	253	193	138
Namibia	178	152	114
Niger	475	392	294
Nigeria	899	833	724
Rwanda	227	209	199
Sierra Leone	Nil	Nil	Nil
South Africa	112	98	79
Senegal	284	237	176
Sudan	274	236	181
Tanzania	498	442	373
Togo	386	367	335

Note: This table presents the forecasts for maternal mortality relative to a 10% increase in PHE in the out-of-sample period, 2021 to 2030. “Nil” indicates the non-negative relationship between PHE and mortality in the country in question; therefore, higher PHE does not reduce maternal mortality. The results from 50 percent of the observation were not reported here in a bid to conserve space; it can be provided on request. Moreover, the outcome of the 75 percent observation is better and more reliable for policy recommendation.

**Figure 2:**
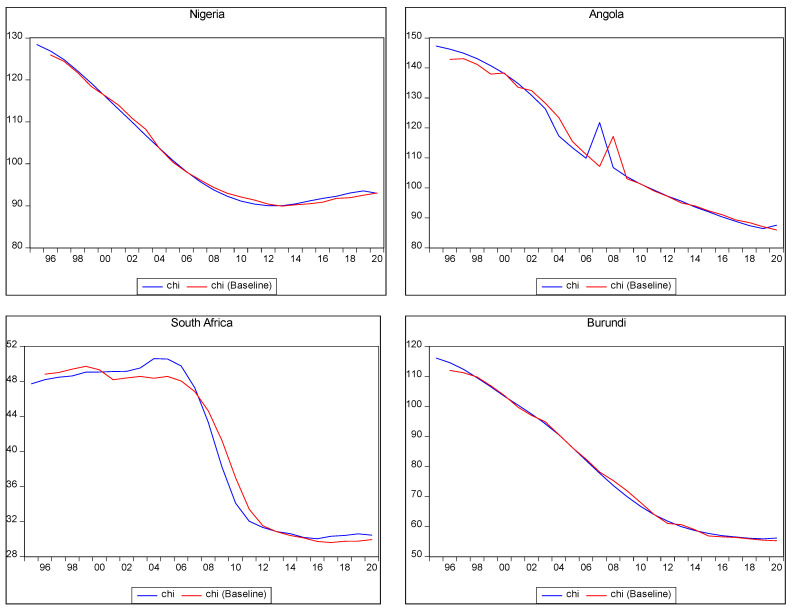

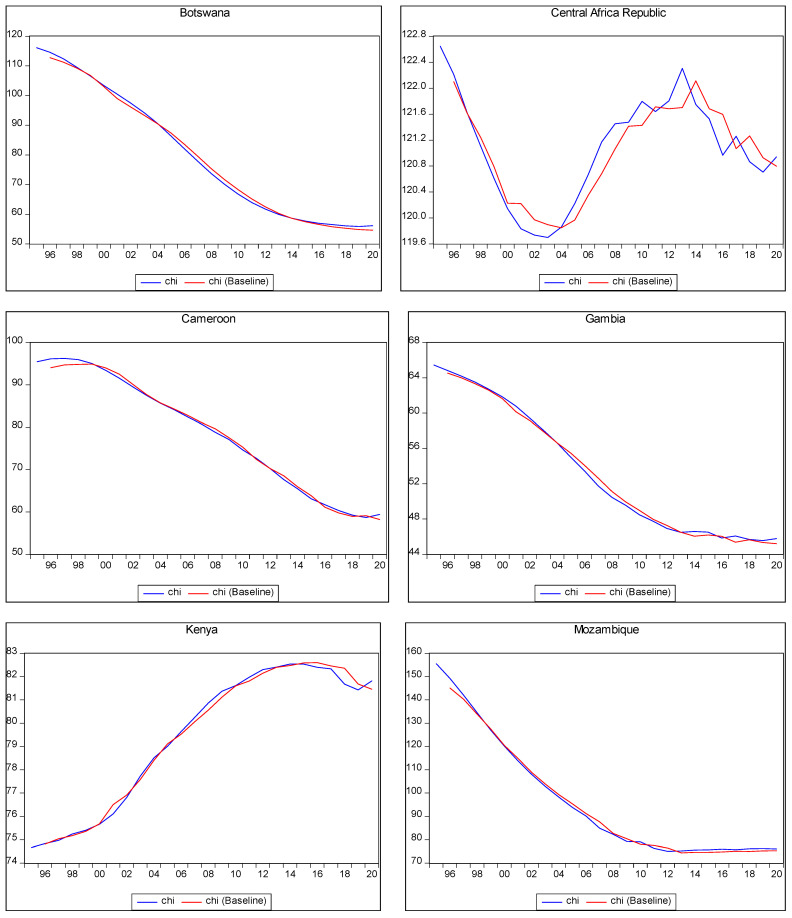

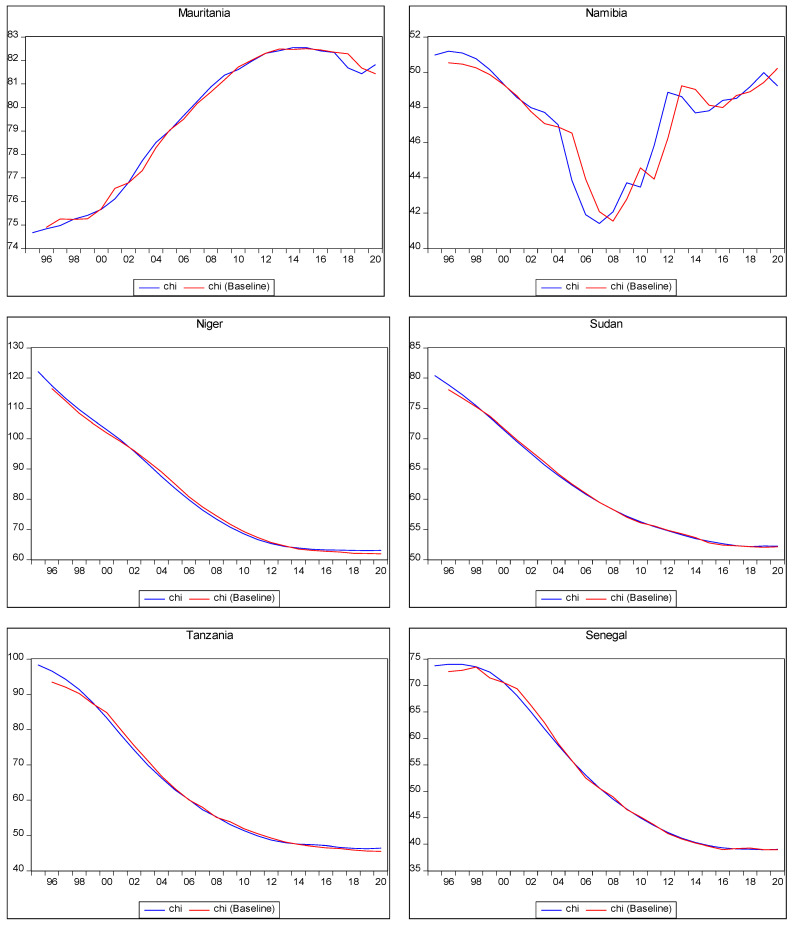

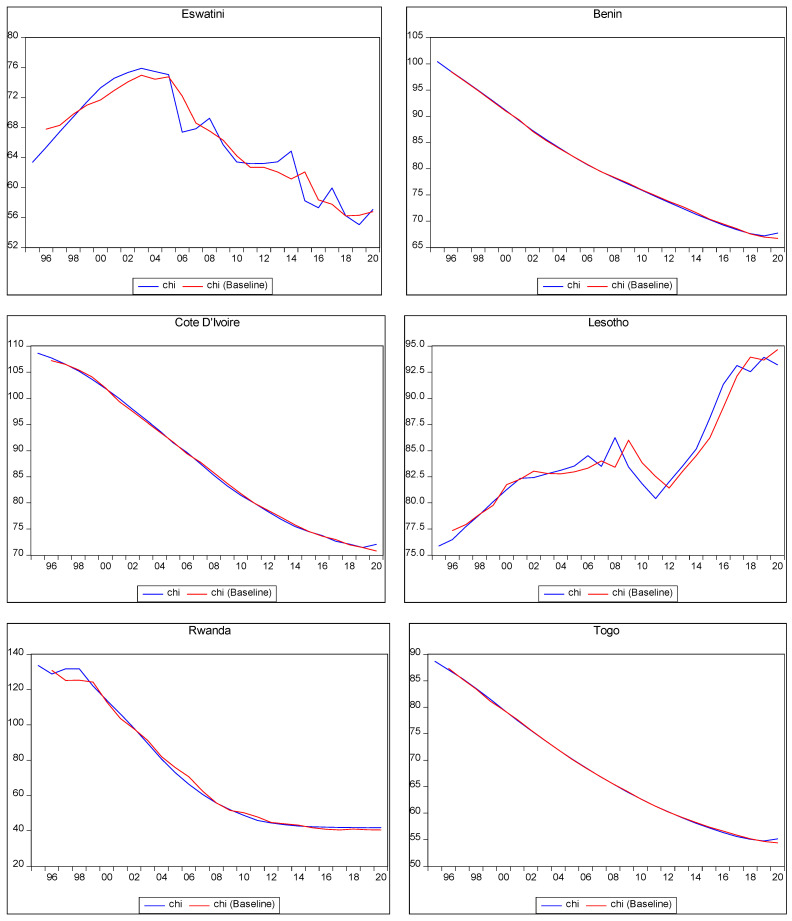

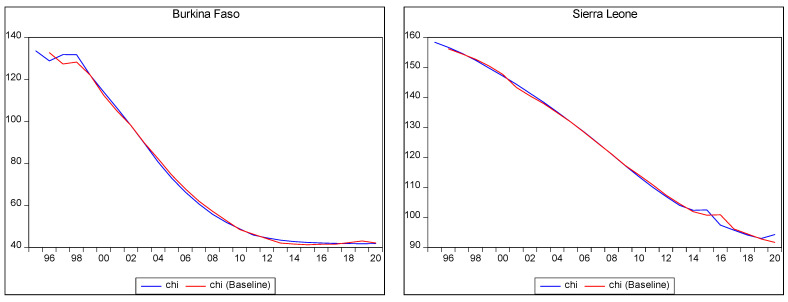
In-sample child mortality prediction.

Figure 2 below displays the in-sample predictions for child mortality. It illustrates the trajectory of actual child mortality and the corresponding forecasted values within the available data period. The alignment between the observed and forecasted child mortality was generally close, with minor variations.

In the figure, the x-axis represents the years, while the y-axis represents child mortality per 1000. The relationship depicted in the figure indicates a downward trend in child mortality over time amongst the countries in SSA. This suggests that child mortality rates decreased during the analysed period.

Despite this declining trend, however, child mortality remained a significant burden in these countries. The reduction in child mortality was positive but insufficient, indicating the need for further efforts to address this issue comprehensively, such as an increase in PHE.

In Figure 2 below, the red curve represents the observed or actual child mortality, while the blue curve represents the forecasted child mortality. The close alignment between these curves indicated the model’s ability to capture the patterns and trends in child mortality within the in-sample data.

The purpose of conducting in-sample predictions is to assess the model’s performance and evaluate its capability to accurately forecast out-of-sample data. In-sample predictions serve as a validation step, ensuring that the model can effectively predict future outcomes beyond the analysed data.

By demonstrating the declining trend in child mortality, the figure highlights the progress made in reducing child mortality rates among the countries in SSA. However, it also underscores the continued challenges and the need for sustained efforts to further reduce child mortality and improve child health outcomes in the region.

Figure 2 below presents the in-sample forecast for child mortality in selected countries in SSA.

Figure 2 above shows the in-sample predictions for child mortality in the selected countries. However, the table below presents out-of-sample predictions of child mortality in a scenario of a 10% increase in the PHE of the countries in SSA during the period from 2021 to 2030. The term “Nil” indicates a non-negative relationship between PHE and child mortality in the respective country, suggesting that higher PHE might not lead to a reduction in child mortality. The table presents the out-of-sample forecasts for child mortality at three specific time points: 2021, 2025, and 2030. The year 2030 aligns with the end of the SDGs timeline when the target is expected to be achieved.

The out-of-sample forecast provides insights into the potential outcomes for child mortality with a 10% increase in PHE. Notably, two countries, South Africa and Senegal, are projected to achieve the SDG target of 25 child deaths per 1000 live births by 2030 if they increase their PHE by 10%. Specifically, with the specified increase in PHE, child mortality is estimated to decrease to 21 in South Africa and 18 in Senegal.

To achieve these outcomes, South Africa would need to increase its PHE per capita to approximately $1558, while Senegal would need to raise its PHE per capita to around $85. These figures highlight the importance of adequate investment in PHE to improve child health outcomes and work towards achieving the SDG target.

The out-of-sample forecasting in Table 3 provides valuable insights into the potential impact of increased PHE on child mortality. It underscores the significance of allocating resources and implementing effective PHE strategies to reduce child mortality rates and progress towards achieving global health goals.

Table 3 below presents the out-of-sample child mortality forecasting, which assumes a yearly 10% increase in current PHE per capita (real terms).

**Table 3:** Out-of-sample child mortality forecasting with a 10% increase in PHE per capita. (Based on 75 percent observation).

Country	2021	2025	2030
Angola	86	82	74
Burkina Faso	42	40	34
Benin	67	65	63
Botswana	54	49	44
Burundi	55	49	40
Cameroon	58	52	40
CAR	121	121	120
Côte d’Ivoire	Nil	Nil	Nil
Eswatini	55	48	39
Gambia	45	43	41
Kenya	82	81	80
Lesotho	93	87	72
Mauritania	83	80	78
Mozambique	75	72	70
Namibia	49	45	34
Niger	61	57	52
Nigeria	92	87	77
Rwanda	40	37	35
Sierra Leone	Nil	Nil	Nil
South Africa	29	26	21
Senegal	38	33	18
Sudan	52	50	47
Tanzania	45	41	36
Togo	54	49	40

Note: This table presents the forecasts for child mortality relative to a 10% increase in PHE in the out-of-sample period, 2021 to 2030. “Nil” indicates the non-negative relationship between PHE and mortality in the country in question. Therefore, a higher PHE would not reduce child mortality.

Table 4 below presents the out-of-sample forecasts for maternal mortality in terms of a 20% increase in PHE during the period from 2021 to 2030.

The table provides insights into the out-of-sample forecast for maternal mortality, specifically considering a 20% increase in PHE. Amongst the countries analysed, only South Africa was projected to achieve the SDG target of 70 maternal deaths per 100,000 by the year 2030. With a 20% increase in PHE, which equates to approximately $3700, the forecasted number of maternal deaths in South Africa by 2030 is estimated to be 62 by 2030. This value of $3700 was derived by multiplying each country’s current PHE by 20%, and subsequent iterations were obtained by multiplying the results from the preceding iteration by an additional 20%. These results underscore the critical role of increased PHE in reducing maternal mortality.

Table 4 below presents the out-of-sample maternal mortality forecasting, which assumes a yearly 20% increase in current PHE per capita (real terms).

**Table 4:** Out-of-sample maternal mortality forecast with a 20% yearly increase in PHE. (Based on 75 percent observation).

Country	2021	2025	2030
Angola	219	171	118
Burkina Faso	281	235	186
Benin	351	298	240
Botswana	128	104	82
Burundi	512	435	332
Cameroon	501	438	361
CAR	770	679	584
Côte d’Ivoire	578	505	421
Eswatini	412	373	317
Gambia	230	198	164
Kenya	325	254	174
Lesotho	527	430	323
Mauritania	734	666	592
Mozambique	242	171	112
Namibia	169	134	91
Niger	460	353	244
Nigeria	875	756	610
Rwanda	217	186	164
Sierra Leone	Nil	Nil	Nil
South Africa	104	84	62
Senegal	275	211	144
Sudan	263	210	148
Tanzania	482	399	312
Togo	373	330	279

Note: This table presents the forecasts for maternal mortality relative to a 20% increase in PHE in the out-of-sample period, 2021 to 2030. “Nil” indicates the non-negative relationship between PHE and mortality in the country in question. Therefore, higher PHE would not reduce maternal mortality.

Table 5 below presents the out-of-sample forecasts for child mortality in terms of a 20% increase in PHE during the period from 2021 to 2030. The designation “Nil” indicates a non-negative relationship between PHE and mortality in the respective country, implying that higher PHE would not result in a reduction in child mortality. However, by the year 2030, the following countries in SSA were projected to achieve the SDG target for child mortality through a 20% increase in PHE: Burkina Faso, Burundi, Cameroon, Eswatini, Gambia, Namibia, Rwanda, South Africa, Senegal, and Tanzania.

In Sierra Leone, an increase in PHE may not have a significant impact on child mortality. This is due to the country’s fragile health system, which was adversely affected by the Ebola epidemic from 2014 to 2016. Additionally, a substantial portion of the PHE in Sierra Leone is financed through out-of-pocket spending (62%), which can limit the potential impact of increased PHE [27]. These forecasted outcomes shed light on the potential effects of increased PHE on child mortality reduction, such as the state of the health system and financing mechanisms, which need to be considered. Table 5 below presents the out-of-sample child mortality forecasting, which assumes a yearly 20% increase in current PHE per capita (real terms).

**Table 5:** Out-of-sample child mortality forecast with a 20% yearly increase in PHE. (Based on 75 percent observation).

Child Mortality	2021	2025	2030
Angola	79	69	57
Burkina Faso	39	31	24
Benin	60	54	48
Botswana	47	39	30
Burundi	48	39	25
Cameroon	50	39	25
CAR	110	105	94
Côte d’Ivoire	Nil	Nil	Nil
Eswatini	46	36	25
Gambia	39	31	24
Kenya	75	68	60
Lesotho	84	73	55
Mauritania	76	68	60
Mozambique	68	60	53
Namibia	42	35	22
Niger	54	47	39
Nigeria	85	74	60
Rwanda	34	28	23
Sierra Leone	Nil	Nil	Nil
South Africa	23	18	12
Senegal	32	25	15
Sudan	45	40	33
Tanzania	39	32	25
Togo	47	39	28

Note: This table presents the forecasts for child mortality relative to a 20% increase in PHE in the out-of-sample period, 2021 to 2030. “Nil” indicates the non-negative relationship between PHE and mortality in the country in question. Therefore, higher PHE would not reduce child mortality.

Table 6 below presents the forecasts for maternal mortality relative to a 30% increase in PHE in the out-of-sample period, 2021 to 2030. “Nil” indicates a non-negative relationship between PHE and maternal mortality in the country in question, which suggests that higher PHE would not reduce maternal mortality. However, the table shows that despite increasing PHE by 30%, only Botswana, Namibia, and South Africa would be able to achieve the SDG target of 70 maternal deaths per 100,000 by 2030. In addition, Burundi, Cameroon, Central African Republic, Côte d’ Ivoire, Eswatini, Lesotho, Mauritania, Niger, Nigeria, Tanzania, and Togo might experience more than 200 maternal deaths before 2030, and Nigeria would still have more than 500.

These forecasted outcomes shed light on the potential effects of increased PHE on maternal mortality reduction. However, specific contextual factors, such as the state of the health system and financing mechanisms, need to be considered. The results align with the study of [16] on the Rwandan economy and [17].

Table 6 below presents the out-of-sample maternal mortality forecasting, which assumes a yearly 30% increase in current PHE per capita (real terms).

**Table 6:** Out-of-sample maternal mortality forecast with a 30% increase in PHE. (Based on 75 percent observation).

Country	2021	2025	2030
Angola	209	150	94
Burkina Faso	270	209	152
Benin	338	267	198
Botswana	120	89	59
Burundi	496	389	276
Cameroon	485	390	301
CAR	748	615	490
Côte d’Ivoire	560	456	352
Eswatini	392	335	263
Gambia	220	175	133
Kenya	313	226	142
Lesotho	510	387	268
Mauritania	692	550	420
Mozambique	231	150	89
Namibia	160	117	70
Niger	445	317	201
Nigeria	851	685	512
Rwanda	207	184	133
Sierra Leone	Nil	Nil	Nil
South Africa	96	71	46
Senegal	262	187	116
Sudan	252	186	120
Tanzania	466	359	259
Togo	360	296	231

Note: This table presents the forecasts for maternal mortality relative to a 30% increase in PHE in the out-of-sample period, 2021 to 2030. “Nil” indicates the non-negative relationship between PHE and mortality in the country in question. Therefore, higher PHE would not reduce maternal mortality.

Table 7 below presents the forecasts for child mortality relative to a 30% increase in PHE in the out-of-sample period, 2021 to 2030. “Nil” indicates the non-negative relationship between PHE and child mortality in the country in question, which indicates that higher PHE would not reduce child mortality. However, the table reflects that with a 30% increase in PHE, the following countries would achieve the SDG target of 25 child deaths per year by 2030: Burkina Faso, Botswana, Burundi, Cameroon, Eswatini, Gambia, Namibia, Rwanda, South Africa, Senegal, Sudan, Tanzania, and Togo. Thus, about 60% of the countries in SSA would be able to achieve the SDG target for child mortality by 2030.

These forecasted outcomes shed light on the potential effects of increased PHE on maternal mortality reduction. However, specific contextual factors, such as the state of the health system and financing mechanisms, need to be considered.

Table 7 below presents the out-of-sample child mortality forecasting, which assumes a yearly 30% increase in current PHE per capita (real terms).

**Table 7:** Out-of-sample child mortality forecast with a 30% increase in PHE. (Based on 75 percent observation).

Country	2021	2025	2030
Angola	72	57	42
Burkina Faso	30	22	13
Benin	53	43	35
Botswana	40	30	21
Burundi	41	30	18
Cameroon	44	32	18
CAR	105	90	75
Côte d’Ivoire	Nil	Nil	Nil
Eswatini	41	29	18
Gambia	33	25	17
Kenya	68	57	46
Lesotho	79	62	41
Mauritania	69	56	46
Mozambique	61	49	40
Namibia	36	27	13
Niger	47	37	27
Nigeria	78	62	45
Rwanda	28	20	14
Sierra Leone	Nil	Nil	Nil
South Africa	21	16	10
Senegal	30	21	9
Sudan	43	39	25
Tanzania	38	27	19
Togo	44	38	23

Note: This table presents the forecasts for child mortality relative to a 30% increase in PHE in the out-of-sample period, 2021 to 2030. “Nil” indicates the non-negative relationship between PHE and mortality in the country in question. Therefore, higher PHE would not reduce child mortality.

## 5. Discussion

Despite the various attempts by African leaders to increase PHE for better health outcomes, the health outcomes in SSA remain poor, with inadequate PHE. The severity of health outcomes in SSA calls for urgent attention to resolve the prevailing health emergency in the region. This study established a predictive link between health outcomes and PHE. The analysis tested the research hypothesis that 10%, 20%, and 30% increases in PHE may not accomplish the SDG target based on the various outcomes of the simulation scenario. In addition, the forecast values for the outcomes from the forecasting analysis showed the percentage level of PHE increase required every year to meet the expected child and maternal mortality that would achieve the SDG target.

The Research Methodology Section explained how other factors affecting child and maternal mortality, including PHE, were considered, and how the forecasting was performed. The projection scenario of a 10%, 20%, and 30% increase in PHE was explained and justified in the section on the forecasting procedure. The in-sample forecasting was conducted to assess the model’s ability to accurately predict outcomes based on the available data. In this case, the in-sample forecast provided insights into the model’s performance in estimating maternal mortality within the investigated period. By comparing actual and forecasted values, researchers can evaluate the model’s accuracy and reliability. This assessment helps build confidence in the model’s ability to make meaningful predictions and inform subsequent out-of-sample forecasting, which extends the predictions beyond the available data to estimate future outcomes. The out-of-sample forecasting involves making predictions or projections for periods beyond the available data used to build the forecasting model. It aims to estimate future outcomes based on the relationships and patterns observed in historical data. In the context of maternal and child mortality forecasting, the out-of-sample forecast provided insights into the potential trends and outcomes for maternal and child mortality beyond the analysed data period. It allowed the researcher to explore the expected impacts of changes or interventions, such as a 10% increase in PHE, on maternal and child mortality rates. In this study, by utilising the forecasting model developed using in-sample data, the out-of-sample forecast estimated how maternal and child mortality may evolve in the future when certain conditions or factors change. In this case, the focus was on the effects of increased PHE on maternal and child mortality rates.

The model specified that the addition of 10%, 20%, and 30% on the current level of spending was maintained throughout the projection period. In this study, the estimation of the models for the period (1995 to 2020) employed a time-series-forecasting technique. The Feasible Quasi-Generalised Least Squares approach was adopted to examine the causal relationship between per capita PHE and health outcomes, with a focus on the expected negative sign indicated by theory whereby higher PHE is expected to result in lower mortality. The chapter explained how, in the study, the estimated results were then used for in-sample and out-of-sample forecasting, including an analysis of an optimistic scenario that examined the likely impact of consistent growth in public expenditure by approximately 10%, 20%, and 30%. Consequently, in the out-of-sample forecasting analysis period, 2021 to 2030, reductions in mortality across the SSA countries were observed. The result implies the need to increase PHE for a reduction in maternal and child mortality in SSA; the simulation result correlates with [16], forecasting results for Rwanda and the [12,13] for OECD countries.

The forecast results indicated a need for a 30% increase in PHE to achieve the SDG target of 70 maternal deaths by 2030 for countries such as Botswana and Namibia. South Africa will achieve the maternal target with just a 20 percent increase in PHE, while countries like Burundi, Cameroon, Central African Republic, Côte d’Ivoire, Eswatini, Lesotho, Mauritania, Niger, Nigeria, Tanzania, and Togo might still experience more than 200 maternal before 2030. According to the forecast, Nigeria might still have more than 500 maternal deaths before 2030, even with a 30% increase in PHE. This outcome underscores the low and inadequate PHE in these countries, aligning with the study conducted by [5], which predicted an increase in PHE for the achievement of UHC in low- and middle-income countries. However, in the current study, about 60% of the countries in SSA were projected to achieve the SDG target for child mortality by 2030 with a 30% increase in PHE.

The results suggested the need for improvement in the level of PHE based on each country’s needs to position the region for achieving the UN 2030 target. The projection results highlighted the need for significant policy challenges in SSA, including addressing wasteful spending, corrupt practices, and inadequate PHE, while improving the depth and quality of healthcare delivery. Based on the analysis, governments should steadily increase PHE beyond the forecast level to improve maternal and child mortality, as current PHE in the region is low and insufficient for achieving the SDG Targets.

The forecasting model’s results corroborated the inadequacy of PHE in SSA, which averaged 1.9% of GNP from 2000 to 2020 [6], with the most recent PHE standing at 2.02% of GNP. In the previous studies [1], a minimum threshold of PHE was estimated to achieve better health outcomes for SSA countries at 3.65% of GNP, indicating that the region is still far from reaching the minimum threshold. Furthermore, the forecasted levels of maternal and child mortality due to a 10%, 20%, and 30% increase in PHE revealed that SSA must strive hard to achieve the SDG targets of 70 maternal deaths per 100,000 births and 25 child deaths per 1000 live births by 2030.

Although the results were insightful, the limitations of the study need, however, to be acknowledged. One such limitation is the constrained study period, which only covered the periods from 1995 to 2020. Extending the study period beyond these years would allow for more robust analysis and improved projections. Additionally, the inclusion of additional predictors could enhance the accuracy and reliability of the predictions or projections. By expanding the temporal scope and incorporating more relevant variables, future research might provide a more comprehensive and nuanced understanding of the subject matter by exploring the non-financial interventions on health outcomes

The results presented in this study, nevertheless, highlighted the urgent need to address the poor health outcomes and inadequate PHE in SSA. The forecasts indicated that current levels of PHE were insufficient to achieve the SDG targets for maternal and child mortality. Steady increase in PHE, based on each country’s specific needs, is needed for the region to progress towards the UN 2030 goal. Therefore, policymakers should prioritise allocating more resources to the health sector and addressing issues, such as wasteful spending and corruption. By improving the depth and quality of healthcare delivery and increasing PHE, SSA could work towards achieving better health outcomes and ultimately realising the SDG health goals. More importantly, a case of emergency must be declared against maternal mortality in SSA.

## 6. Conclusions and Recommendations

### 6.1. Conclusions

In conclusion, maternal and child mortalities were forecasted in relation to 10, 20, and 30 percent increases in public health expenditure per capita with the aim of achieving the SDG target of less than 70 and 25 deaths per 100,000 and 1000 live births, respectively. The time-series-forecasting technique was employed in the estimation of the models (1995–2020). The Feasible Quasi-Generalised Least Squares approach was adopted to investigate the causal relationship between public health expenditure per capita and the health outcomes. The in-sample results validate the out-of-sample results [28,29,30], and the simulation results indicate that a 30 percent increase in public health expenditure will achieve an SDG target of less than 70 maternal deaths in Botswana, Namibia, and South Africa, and 60 percent of SSA countries will achieve 25 child deaths by year 2030.

### 6.2. Recommendations

The results suggest the need for improvement in the level of PHE based on each country’s needs to position the region to achieve the UN 2030 targets. The projection results highlighted the need for significant policy challenges in SSA, including addressing wasteful spending, corrupt practices, and inadequate PHE, while improving the depth and quality of healthcare delivery. Based on the analysis, governments should steadily increase PHE beyond the forecast level to improve maternal and child mortality rates, as the current PHE in the region is low and insufficient to achieve the SDG targets. Additionally, the government should consider policies beyond increasing PHE, such as improving healthcare infrastructure and training healthcare workers.

## Figures and Tables

**Table 1:** Regression results (relationship between health outcomes and PHE per capita).

Country	Maternal Mortality Model		Child Mortality Model	
	Constant	Beta	Constant	Beta
Angola	0.3833 ** (0.1802)	−0.0487 * (0.0253)	0.3417 (0.2855)	−0.0241 (0.0267)
Burkina Faso	0.2350 * (0.1289)	−0.0211 (0.0173)	−0.4235 ** (0.1971)	−0.0345 (0.0318)
Benin	−0.1024 (0.2296)	−0.0073 (0.0086)	0.6212 ** (0.2238)	−0.00003 (0.0042)
Botswana	0.1998 (0.3042)	−0.0134 (0.0147)	0.3634 * (0.1765)	−0.0176 * (0.0098)
Burundi	0.3571 ** (0.1396)	−0.0444 ** (0.0223)	0.2629 *** (0.0584)	−0.0611 *** (0.0132)
Cameroon	−0.0474 (0.1526)	−0.0169 (0.0122)	−0.3046 *** (0.0776)	−0.0288 *** (0.0077)
CAR	0.8856 (0.6401)	−0.0057 (0.0182)	0.7619 ** (0.3513)	−0.0013 ** (0.0005)
Côte d’Ivoire	0.0470 (0.2947)	−0.0107 ** (0.0049)	0.0828 *** (0.0332)	0.0162 *** (0.0032)
Eswatini	1.3503 *** (0.2642)	−0.0444 *** (0.0084)	3.1321 *** (0.8269)	−0.0625 *** (0.0149)
Gambia	0.2151 (0.1953)	−0.0107 (0.0076)	0.4274 *** (0.1272)	−0.0146 ** (0.0054)
Kenya	0.9180 ** (0.3985)	−0.0905 (0.0666)	0.2957 *** (0.0680)	−0.0055 *** (0.0010)
Lesotho	1.7660 ** (0.6734)	−0.0818 *** (0.0288)	0.7056 (0.4219)	−0.0111 (0.0155)
Mauritania	0.3311 (0.2511)	−0.0074 ** (0.0029)	0.2069 *** (0.0673)	−0.0094 *** (0.0019)
Mozambique	5.0635 (1.0313)	−0.0006 (0.0061)	0.3215 *** (0.0708)	−0.0003 (0.0076)
Namibia	0.5101 (0.4279)	−0.0596 (0.0371)	0.3195 (0.3019)	−0.0966 * (0.0475)
Niger	2.1183 *** (0.6618)	−0.0117 * (0.0062)	0.2292 *** (0.0541)	−0.0133 (0.0136)
Nigeria	0.5917 *** (0.0948)	−0.0380 *** (0.0074)	0.1329 * (0.0665)	−0.0328 *** (0.0066)
Rwanda	1.2979 ** (0.4721)	−0.0357 (0.0449)	1.0869 *** (0.3450)	−0.0075 (0.0286)
Sierra Leone	0.3608 *** (0.1152)	0.0343 *** (0.0074)	0.0601 (0.0599)	0.0098 *** (0.0032)
South Africa	0.4511 (0.3919)	−0.0364 * (0.0204)	1.1223 *** (0.3392)	−0.0838 *** (0.0233)
Senegal	−0.0670 (0.1615)	−0.0216 (0.0378)	−0.2318 *** (0.0465)	−0.1414 *** (0.0173)
Sudan	0.4719 ** (0.2007)	−0.0559 *** (0.0184)	0.2571 *** (0.0285)	−0.0159 *** (0.0035)
Tanzania	2.3167 ** (0.6448)	−0.0068 (0.0076)	0.2109 *** (0.0498)	−0.0270 ** (0.0118)
Togo	0.0974 (0.1718)	−0.0137 * (0.0067)	0.7327 *** (0.2506)	−0.0061 * (0.0033)

Note: ***, **, and * signify 1%, 5%, and 10% significance, respectively.

## Data Availability

The data adopted in the study are available in public domains and can be provided on request. Available at https://apps.who.int/nha/database/select/indicators/en (accessed on 6 January 2024).
